# A Case of Perforation of the Intestine

**Published:** 1864-05

**Authors:** W. D. Slayter

**Affiliations:** Chicago, Graduate Royal College of Physicians of England, Royal College of Surgeons of London and Dublin. Late House-Surgeon of Westminster Hospital, London


### A CASE OF PERFORATION OF THE INTESTINE.

By W. D. SLAYTER, M. D., of Chicago,

*Graduate Royal College of Physicians of England, Royal College of Surgeons of London and Dublin. Late House-Surgeon of Westminster Hospital, London.*

The following case, I think, will be of some interest to the profession, as showing, first, how very slight the symptoms, in some cases of abdominal injury, are in comparison to the extent of the injury received; second, how guarded the surgeon ought always to be in making his diagnosis and prognosis in such cases. In this case almost the only symptom present which pointed to some very extensive injury was, the state of the pulse—it ranging from 100 to 140 beats in the minute.

At first, probably, there was merely contusion of the intestine, but subsequently ulceration and perforation took place, which caused death on the ninth day from the receipt of the injury. Without any further remarks, I will give a brief outline of the case; and it will be seen that it was impossible to form any positive diagnosis from the symptoms present.

Dudley Wells, æt. 36, admitted into the accident ward of the Westminster Hospital June 18th, 1861. His friends stated that when returning from the Volunteer Rifle Review in Hyde Park, and while crossing Westminster Bridge, his horse took fright, reared, and fell backwards, the rider being under the animal, and the pommel of the saddle striking him in the abdomen. He was immediately carried to the Hospital, and with the assistance of a friend walked from the Surgery to the Ward, a distance of fifteen or twenty yards. When I saw him on admission I found him suffering from some mental excitement, perspiring freely, skin very hot, pulse quick; but there was no pain or prostration present. On attempting to pass urine, he found that he was unable to do so; a catheter was passed and nearly a pint of clear urine drawn off, showing, that although the abdominal muscles had been paralyzed by the shock, still the bladder itself had not been ruptured.

On further examination there was found an extensive bruise of the lower part of the abdomen, extending from one iliac process to the other, attended with considerable pain on pressure. He was ordered to take ʒj of the liq. opii sed., and to have hot flannels sprinkled with tinct. opii applied to the part. This treatment relieved him very much, and he slept well for some hours, but as he was unable to void his urine, a catheter was passed twice a day.

For two days he progressed favorably, but on the morning of the third he woke up complaining of violent twisting pain in the right side, accompanied by great tenderness—pulse 135, and small. A 5 gr. pil. sapon. com. was administered, which relieved him very much. For five days he was kept constantly under the influence of opium, but the pulse continued to range from 130 to 140, and very small and thready.

On the ninth day he complained of excruciating pain in the same situation; another dose of opium relieved him, but about six, P. M., on turning in bed very suddenly, he felt something give way internally, immediately alarming symptoms of prostration came on, and four hours afterwards he was dead.

Throughout the whole time he took beef tea, brandy and egg, etc., but the bowels were not once moved, nor did they seem at all inclined to act, and as it was evident some serious injury had been done to the intestine, it was deemed imprudent to attempt to disturb them.

A post-mortem examination was made twenty-four hours after death, when a hole about the size of a silver quarter of a dollar, with clean cut edges, was found in the jejunum, one foot from the duodenum; lymph had been thrown out around the ulcer, causing that part to adhere to the other intestines.

All the other viscera were healthy; the inflammation seemed to be confined to the immediate neighborhood of the sore. The sudden turning in bed on the 9th day, I presume, tore apart the adhesions and this caused death from prostration.

